# Combining magnetic forces for contactless manipulation of fluids in microelectrode-microfluidic systems

**DOI:** 10.1038/s41598-019-41284-0

**Published:** 2019-03-25

**Authors:** Veronika Haehnel, Foysal Z. Khan, Gerd Mutschke, Christian Cierpka, Margitta Uhlemann, Ingrid Fritsch

**Affiliations:** 1 Institute for Complex Materials, IFW Dresden, Helmholtzstr. 20, D-01069 Dresden, Germany; 2 Department of Chemistry and Biochemistry, University of Arkansas, Fayetteville, AR 72701 USA; 3 Helmholtz-Zentrum Dresden-Rossendorf, Bautzner Landstr. 400, D-01328 Dresden, Germany; 4 Institute of Thermodynamics and Fluid Mechanics,Technische Universität Ilmenau, D-98684 Ilmenau, Germany

## Abstract

A novel method to drive and manipulate fluid in a contactless way in a microelectrode-microfluidic system is demonstrated by combining the Lorentz and magnetic field gradient forces. The method is based on the redox-reaction [Fe(CN)_6_]^3−^/[Fe(CN)_6_]^4−^ performed in a magnetic field oriented perpendicular to the ionic current that crosses the gap between two arrays of oppositely polarized microelectrodes, generating a magnetohydrodynamic flow. Additionally, a movable magnetized CoFe micro-strip is placed at different positions beneath the gap. In this region, the magnetic flux density is changed locally and a strong magnetic field gradient is formed. The redox-reaction changes the magnetic susceptibility of the electrolyte near the electrodes, and the resulting magnetic field gradient exerts a force on the fluid, which leads to a deflection of the Lorentz force-driven main flow. Particle Image Velocity measurements and numerical simulations demonstrate that by combining the two magnetic forces, the flow is not only redirected, but also a local change of concentration of paramagnetic species is realized.

## Introduction

New technologies based on transport, mixing, actuation and manipulation of fluids and objects in the micro- and nanometer scale are rapidly developing. The enormous scientific and technological interest focuses on total microanalysis approaches which are applicable in analytics and monitoring in medicine, biology, and the environment. Contactless external driving forces such as electric or magnetic fields and field gradients for tailored fluid manipulation and electrochemical and analytical approaches are of increasing interest. Most of them are focused on magnetic fluids containing magnetic or superparamagnetic particles ranging from the nanoscale to the microscale allowing pumping^1^, manipulation^2^, mixing^3^, sorting or trapping^4,5^. The mixing of two fluids can be considerably accelerated and controlled by superparamagnetic microbeads superimposed to pulsed magnetic fields^6,7^ or rotating magnetic fields^5^. Recently, comprehensive reviews of the application of ferrofluids for micro-magnetofluidic applications have been published^8–10^. A new method for trapping of biological cells by implementing paramagnetic structures in microfluidic channels has been reported^11^. Another branch deals with weak electrically conducting fluids where pumping and mixing can be achieved by superimposing *both* magnetic and electric fields based on the magnetohydrodynamics (MHD) effect^12^. First investigations to increase fluid velocities in microchannels by DC- and AC-MHD micropumping were reported more than 10 years ago^13,14^. Potentials applied at well-designed electrodes must not be too extreme so that bubble formation from water electrolysis in aqueous electrolytes and electrode corrosion can be avoided. This holds also for electrokinetic methods applied for pumping on the microscale^14,15^. To overcome this disadvantage two concepts have been described. One uses an AC electric field which requires electromagnets and synchronized operation conditions to obtain unidirectional flow^16^. A second way involves reversible redox couples to enable sufficient ionic currents with low overpotential which also prevents electrode degradation and heat generation, called redox**-**MHD^17–22^. Many studies dealing with redox-MHD have shown that a small fluid volume can be driven in confined spaces. An electronic current applied to electrodes produces an ionic current across the gap between the anode and cathode, which can be strategically arranged on microchips. Well-known redox species are [Fe(CN)_6_]^4−^/[Fe(CN)_6_]^3−^ converting into each other by generating and consuming one electron at the anode and cathode, respectively. That provides a continuous flow over an indefinite time, as the reacting ions are not exhausted, and, for electrode geometries like parallel bands, fresh solution consistently enters one end and reacted solution exits the other end of the stream. The ionic current density **j** (A/m²), and the magnetic flux density **B** (T) directed perpendicular to the plane containing the electrodes, generate by the right hand rule, a net Lorentz force **f**_**L**_ *(N/m³*) parallel to the chip surface^23,24^

1 $${{\rm{f}}}_{{\rm{L}}}={\rm{j}}\times {\rm{B}}{\rm{.}}$$

It has been shown that by changing the external magnetic field locally the resulting Lorentz force changes and influences the fluid flow^25^. Therefore, redox-MHD is very unique compared to mechanical and electrokinetic methods^8^, as well as to the utilization of ferrofluids. As it does not need any moving parts, it allows flexibility in device design. It offers multidirectional and channel-less fluid flow^19^ and compatibility with both aqueous and non-aqueous solutions. The electrode reactions can also produce gradients of mass density and electrical conductivity^26^ that result in additional forces which may further influence the fluid flow.

Another intriguing, but little investigated method to influence fluid flow arises from applying magnetic field gradients^27,28^. Those produced from magnetized ferromagnetic tracks can act like a magnetic channel and stabilize paramagnetic liquids^29,30^. Enrichment and fluid flow have been demonstrated for paramagnetic ions in high magnetic field gradients^31,32^. In the absence of electric fields and electric currents and if paramagnetic ions and inhomogeneous magnetic fields are involved, the magnetic field gradient force (**f**_∇B_)^33,34^ may change the concentration of paramagnetic species locally. **f**_**∇B**_ depends on the magnetic flux density **B**, its gradient **∇B**, the molar magnetic susceptibility *χ*_mol,k_ and the concentration *c*_k_ of every species *k* in the electrolyte, according to

2 $${{\bf{f}}}_{{\rm{\nabla }}{\rm{B}}}=\frac{{\chi }_{sol}}{{\mu }_{0}}({\bf{B}}\cdot {\rm{\nabla }}){\bf{B}}\,with\,{\chi }_{sol}={\sum }_{k}{\chi }_{mol,k}{c}_{k+}{\chi }_{{H}_{2}O}.$$

In addition to the magnetic field gradient a gradient of concentration is formed by the electrochemical processes at the electrodes^35^. Then the rotational part of the equation can drive fluid flow which influences the fluid velocity near the wall or the electrodes^33^.

3 $$\nabla \times {{\bf{f}}}_{\nabla {\rm{B}}}=\frac{1}{2{\mu }_{0}}({\sum }_{k}{\chi }_{mol,k}\nabla {c}_{k})\times ({\nabla }{B}^{2})$$

It was demonstrated earlier, that this can be utilized for producing patterned and structured electrodeposits (millimeters)^35,36^. In summary, two magnetic forces, **f**_**L**_ and **f**_∇B_, are available for driving and manipulating fluid flow at small scales.

The study herein investigates the effects of both the magnetic fields and their gradients in modifying the fluid flow in a cell at even smaller dimensions (involving tens of micrometers) that are of interest in the field of microfluidics. The ionic current is produced from the redox reactions of [Fe(CN)_6_]^3−^ and [Fe(CN)_6_]^4−^ at two arrays of microelectrodes separated by 550 µm. Strong magnetic field gradients are obtained from a 50 µm magnetized ferromagnetic CoFe strip placed beneath the cell, and which are expected to attract fluid with the paramagnetic [Fe(CN)_6_]^−3^ ions.

The question addressed here is whether, in addition to the well-studied Lorentz force, the magnetic gradient force can be used to control and manipulate the flow and possibly influence the mass transfer. This is a departure from our previous work using this same microfluidic chamber^17,22^, which focused on manipulation of flow by changing the ionic current density by varying electronic activation of the electrodes, instead of modifying the magnetic field.

Particle image velocimetry (PIV) was applied to estimate the velocity and the direction of the resulting fluid flow. In parallel, numerical simulations were performed to analyze the magnetic field distribution, the fluid flow and the species concentration, the Lorentz and the field gradient forces, and comparisons are made with the experimentally obtained results.

## Results and Discussion

**Figure 1:**
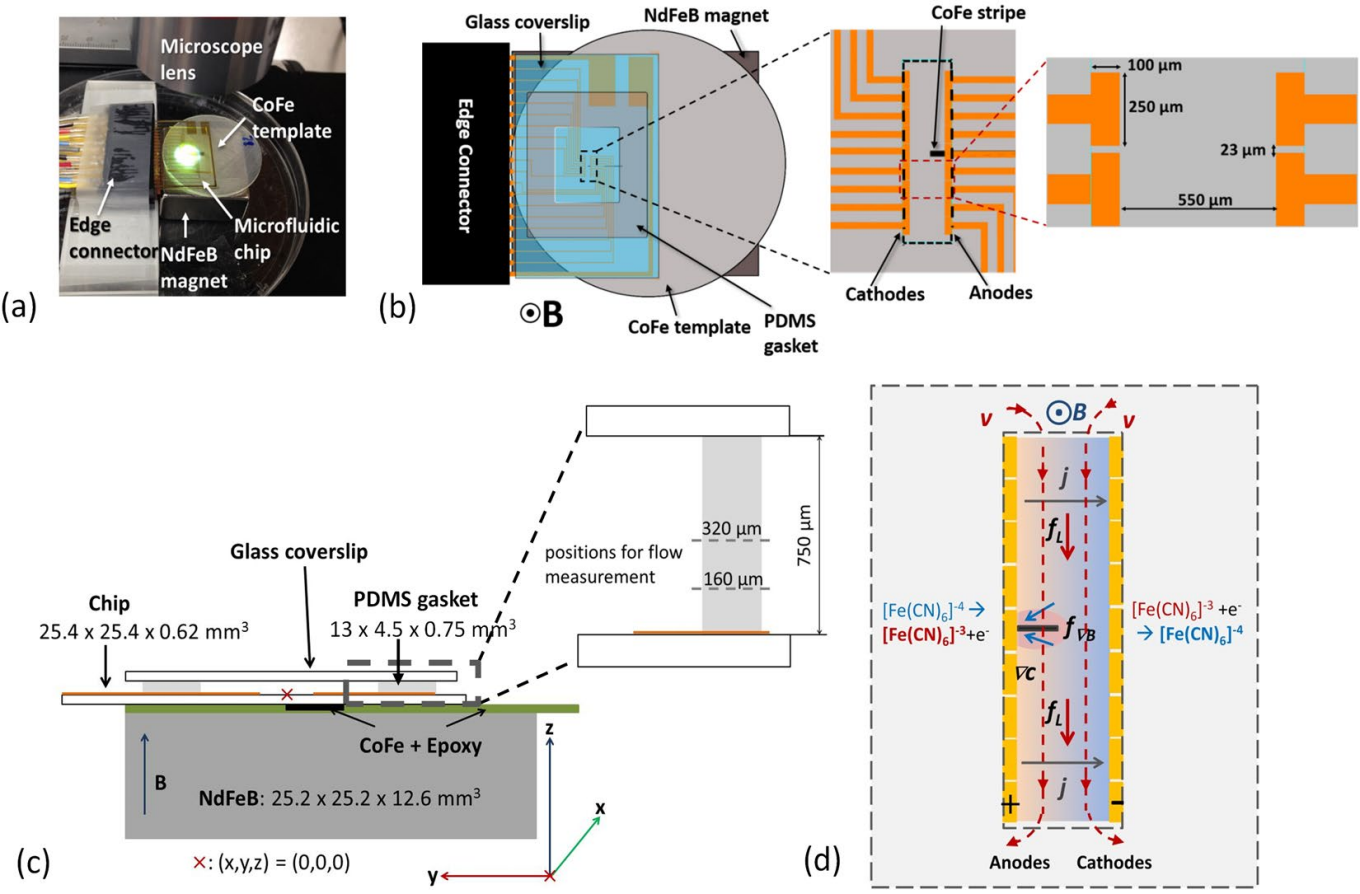
(**a**) Photographic image of the experimental setup. (**b**) Schematic of top-down view of the electrochemical chamber/magnet assembly with edge connector, expanded view of the electrode arrays (orange) with the CoFe-strip (solid black) placed underneath the 5^th^ (middle) electrode on the anode side, and a drawing showing electrode dimensions. Outside the black-dashed square the electrode leads are electrically isolated with benzocyclobutene (BCB). (**c**) Sketch of the side view of the cell design with dimensions. The CoFe strip size is 5 mm × 1 mm × 50 µm. The chip is placed on top of the permanent magnet with the movable CoFe strip embedded in epoxy between them. The cutout within the PDMS forms the chamber that contains the solution that is enclosed on top with a glass coverslip. The magnification shows the z positions for the velocity measurements. (**d**) Sketch of the forces, the electrochemical reactions, the concentration gradients formed at the electrodes and the flow path. The sketch is mirrored axially compared to (**a**–**c**) to represent the flipped orientation seen by the camera through the optics of the microscope and to allow comparison with the subsequent images from measurements and simulations.

The setup shown in Fig. 1(a–c) is used for the experiments. It consists of a microfluidic-microelectrode chip placed on top of a permanent magnet. The magnetic field is directed upward, thus perpendicularly penetrating the flat chip. The electrodes are made of Au and the active area is marked by the dashed rectangle (Fig. 1(b)). A PDMS gasket defines the volume of the cell. The cell is covered by a glass slide to avoid evaporation, as sketched in Fig. 1(c). The origin (x, y, z)=(0, 0, 0) of the coordinate system is located in the center between the electrode arrays on the bottom of the microfluidic chip. Between the arrays of anodes and cathodes an anodic current of −10 µA is applied (Fig. 1(d)). For electrochemical characterization of the electrodes and more details of the electrode chip design see SI Figs S1 and S2, respectively.

From the right hand rule, the Lorentz force is expected to drive a horizontal flow between and parallel to the electrodes. When the flow reaches the end of the electrodes it loops back around the outside of the electrodes and re-enters the opposite side of the electrode array, as sketched in Fig. 1(d). The electrochemical redox-processes form concentration gradients as well as density gradients at the anode and cathode. Diamagnetic [Fe(CN)_6_]^−4^ ions are converted to the paramagnetic [Fe(CN)_6_]^−3^ ions at the anode and vice versa at the cathode. To balance the charge of the electrolyte at the electrodes, these and other ions in solution will move away and toward the electrodes, accordingly, as discussed by Sahore *et al*.^37^. In order to investigate the impact of magnetic field gradients on the fluid flow, a CoFe strip (**gradB**-strip) embedded in epoxy was placed between the permanent magnet and the microfluidic chip. The magnetization of the **gradB**-strip leads to strong magnetic field gradients in its vicinity. The strip is arranged exactly underneath the 5^th^ electrode in the anode array (SI Fig. S2) whereby its position across the electrode gap (y-direction) can be adjusted. The CoFe strip locally causes a higher magnetic field that leads to a stronger Lorentz force. And it has been shown in earlier investigations that the related magnetic gradient force also influences the flow of a fluid which contains paramagnetic ions^27,32,33,35^. Hence, a modification of the fluid flow that is mainly driven by the Lorentz force can be expected which is caused by the ferromagnetic strip underneath.

### Fluid flow – measured by PIV

**Figure 2:**
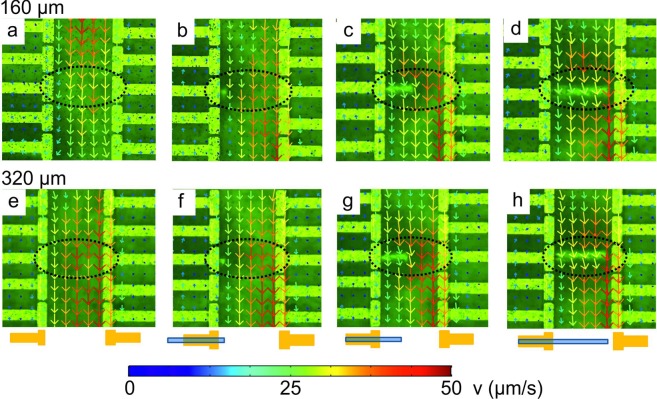
Microscope images of the central section of the electrode array region in the microfluidic chamber. The color and length of the arrows represent the averaged velocity vectors at a height above the electrode chip of (**a**–**d**) z = 160 µm and (**e–h**) z = 320 µm. The dotted oval contours represent the area affected by the **gradB**-strip that is placed beneath the electrode chip at different positions in the y-direction. The images correspond to the five central electrodes of each array in the sketch shown in Fig. 1(d).

Flow profiles were monitored by tracking microbeads and analyzed by applying two different PIV methods described in detail in the Supplementay Information. Figure 2 shows velocity vectors superimposed on photographic images in the xy-plane at a distance of z = 160 µm and z = 320 µm above the bottom of the microfluidic system, respectively. The length and the color of the arrows represent the velocity magnitude given in µm/s. The photographic images are mirrored along the axial dimension, compared to the photographs and sketches in Fig. 1(a–c), to represent the flipped orientation viewed by the camera through the microscope optics. In Fig. 2(a,e) the velocity fields are depicted without the **gradB-**strip in two horizontal xy-planes at z = 160 μm and z = 320 μm height. Despite the rather flat velocity profiles that were obtained across the electrode gap in similar microfluidic redox flow cells^17^, the flow profiles found here show a maximum velocity in the center and have nearly a parabolic shape. The velocity is also slightly higher in the region of the cathode. The reason for this may expected to be a small misalignment of the chip from the horizontal direction when placing the chip, i.e. a tilting in the y-direction. Additionally, the permanent magnet was not perfectly centered below the chip due to geometrical constraints of the setup. The latter geometrical aspect was taken into account in the numerical simulations, which will be discussed below. Figure 2(b,f) illustrate the CoFe strip positioned in the middle of the anode array, under the 5^th^ electrode, and just starting to enter the gap between the arrays. A slightly lower velocity results at the anode just above the **gradB-**strip and a faster velocity at the cathode compared to the original horizontal flow profile. Upon moving the **gradB-**strip more into the gap, further changes of velocity profiles are observed, as shown in Fig. 2(c,g) for the position where the **gradB**-strip reaches the middle of the gap and in Fig. 2(d,h) where the **gradB**-strip almost reaches the cathode side. The direction of the flow above the **gradB**-strip tilts to the cathode and the velocity increases significantly and is lower at the anodes. The maximum velocity measured is about 50 µm/s. Various reasons for the change of the velocity profile in the region of the **gradB**-strip have to be considered. (i) The effect is caused by the small change of the magnitude of **B** resulting in a local change of the Lorentz force ${{\bf{f}}}_{{\bf{L}}}$ (eq. (1)) affecting the MHD flow close to the magnetized CoFe strip. (ii) The field gradient force ${{\bf{f}}}_{{\boldsymbol{\nabla }}{\bf{B}}}$ leads to a deflection from the original flow direction. The [Fe(CN)_6_]^4−^ species oxidize at the anode, producing paramagnetic [Fe(CN)_6_]^3−^ species. According to Eqs 2 and 3 the fluid with the paramagnetic species might be pushed to the region of the **gradB**-strip at the anode where the velocity of the ${{\bf{f}}}_{{\bf{L}}}$ induced main flow is retarded and thus a local, change of concentration occurs near the anodes. This alters locally the distribution of the ionic current density and then in turn impacts ${{\bf{f}}}_{{\bf{L}}}$.

### Numerical simulations

To clarify the impact of the magnetic field gradient on the velocity profile, 3D numerical simulations were performed using the commercial software COMSOL Multiphysics 5.2a. A sketch of the simulation setup with the grid and coordinates is provided in SI Fig. S6. Velocity profiles were obtained where the **gradB**-strip is absent and where it reaches the middle of the gap between the anodes and cathodes, as depicted in Fig. 2(c,g), respectively. The simulations deliver information on **B** and **gradB**, the concentration profile of the two electroactive species, the resulting paramagnetic susceptibility (*χ*_sol_) of the solution, the Lorentz force ${{\bf{f}}}_{{\bf{L}}}$ and field gradient force ${{\bf{f}}}_{{\boldsymbol{\nabla }}{\bf{B}}}$, and the resulting velocity profiles in x, y, and z-directions. For simplicity, the density gradients due to the electrode reactions and the resulting natural convection mentioned before were not considered in the simulations. From the experimental and numerical results it can be estimated that the Reynolds number in this setup (based on the cell height) is much smaller than 1, leading to a velocity profile that spreads horizontally between the electrodes and vertically between the top and the bottom of the cell. The steady state flow regime takes a long time to develop and was not yet achieved at the instant of t = 60 s when measurements were obtained in the experiments. This was taken into account in the simulations such that limiting current boundary conditions were applied but the amplitude of the magnetic gradient force ${{\bf{f}}}_{{\boldsymbol{\nabla }}{\bf{B}}}$ was diminished to 10% in order to account for an overall weaker action of the gradient force.

**Figure 3:**
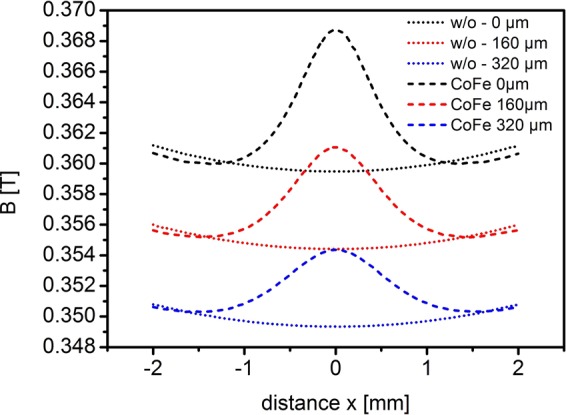
|**B**| distribution along the x direction of the fluidic chamber with the end of the CoFe **gradB-**strip located at x = 0 and y = 0 for 3 different distances above the electrode chip. Dotted lines – without the CoFe **gradB-**strip; dashed lines - with the CoFe **gradB**-strip.

**Figure 4:**
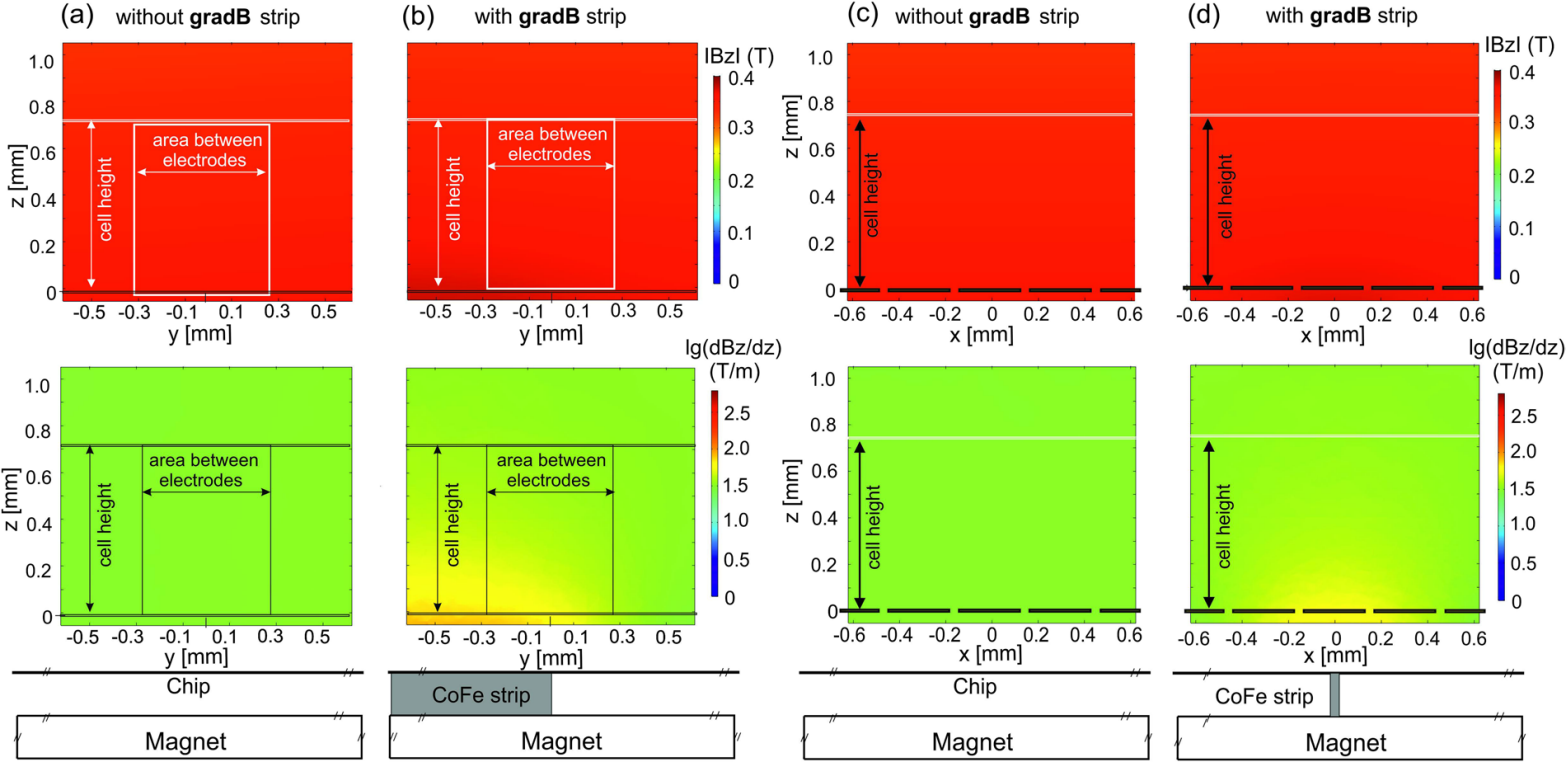
Simulation of IB_z_I and **lg(**dB_z_/dz**)** distribution over the height of the chamber without (**a**,**c**) and with the CoFe strip (**b**,**d**) for cases where (**a**,**b**) the yz vertical plane is at x = 0 and (**c**,**d**) the xz vertical plane is at y = 0.

In Fig. 3, the calculated modulus of **B** along the streamwise (x) direction is shown at different distances from the bottom of the microfluidic chip for the two cases, with and without **gradB-**strip. The CoFe strip is located at the position x = 0. Results are shown for the bottom of the microfluidic chip and at heights of z = 160 µm and z = 320 µm above the bottom. In the center of the **gradB**-strip the magnetic field is slightly enhanced at most by 10 mT and 6 mT for z = 160 µm and z = 320 µm, respectively. Also the change of **|B|** in the z-direction with and without **gradB**-strip is small, shown in Fig. 4. Considering the small changes of **B** and B_z,_ in comparison to the case without the CoFe strip, a negligible distortion effect on ${{\bf{f}}}_{{\bf{L}}}$ is expected. More significant is the change of (dB_z_/dz) with **gradB**-strip, displayed on a logarithmic scale and depicted in Fig. 4(b,d) for the yz- and xz-planes, respectively. In Fig. 4(b,d) (dB_z_/dz) amounts to several 100 T/m at the bottom of the microfluidic chip and is still about 100 T/m at z = 160 µm and z = 320 µm. The magnitude of the magnetic field gradient (B gradB) decays nearly exponentially with the distance between the surface of the **gradB**-strip to the surface of the cell. Since the thickness of the chip bottom was fixed, the CoFe strip was selected to have a sufficiently strong magnetic field gradient, see also SI Fig. S4.

**Figure 5:**
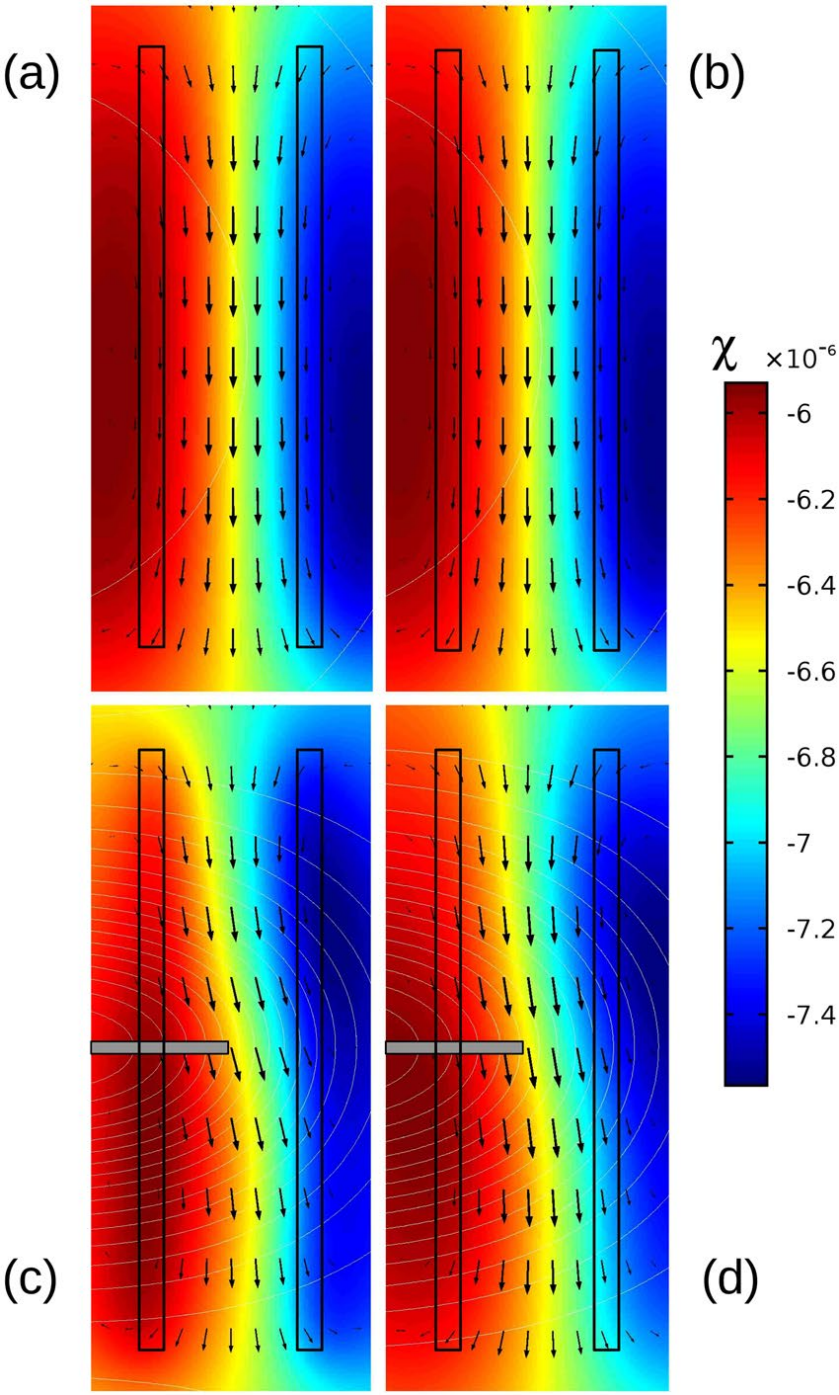
Simulation result of the velocity field (black arrows), the magnetic susceptibility of the fluid (color contours) and the magnitude of **B** (white contour lines) shown at t = 60 s after switching on the cell current, depicted at two horizontal xy-planes of z = 160 µm (left) and z = 320 µm (right). The electrode lines are marked as black rectangles. Top (**a**,**b**) – without, and bottom (**c**,**d**) - with **gradB-**strip.

**Figure 6:**
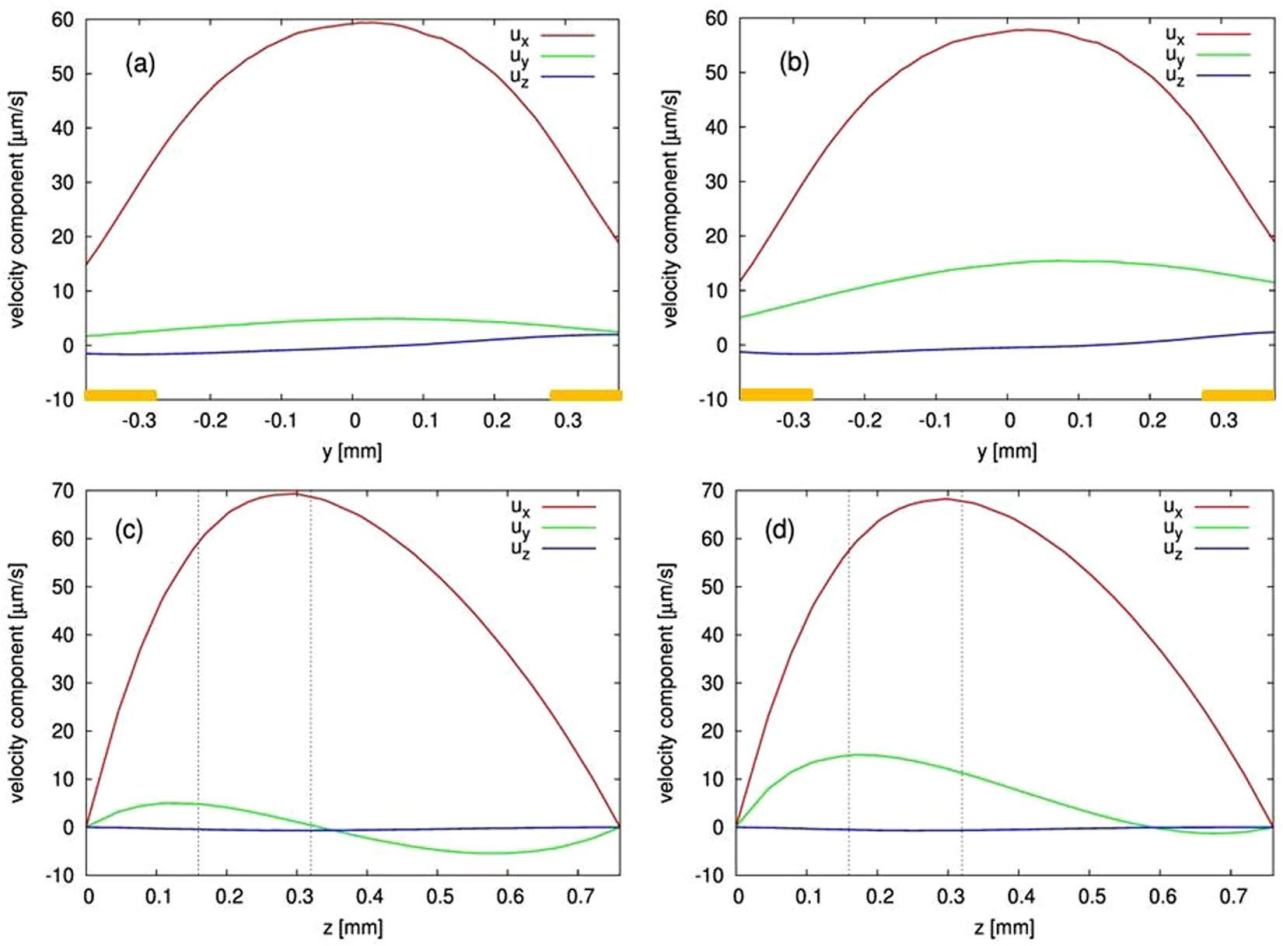
Profiles of the velocity components at x = 0 without (**a**,**c**) and with (**b**,**d**) **gradB**-strip. (**a**,**b**) shows the profile between the electrodes at z = 160 µm. The yellow bars illustrate the position of the electrodes. (**c**,**d**) shows the vertical profile at y = 0. The dashed lines mark the distances z = 160 µm and 320 µm above the bottom of the cell.

**Figure 7:**
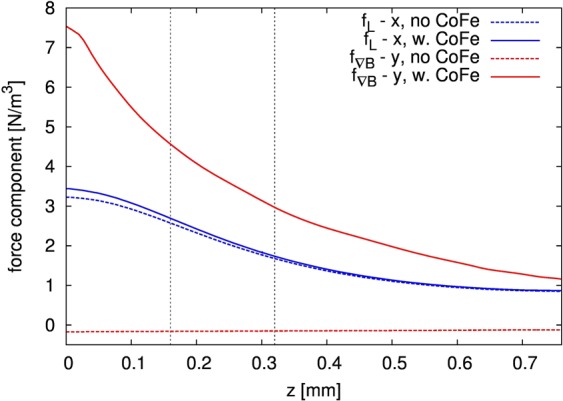
x- component of **f**_**L**_ and y-component of **f**_**∇B**_ at x = 0, y = 0 versus cell height z without and with **gradB**-strip.

To understand the complexity of the different magnetic forces driving the fluid, the following simulation results depict the situation at t = 60 s after switching on a cell current of −10 µA. In Fig. 5, the velocity profile marked by black arrows, contour lines of the magnetic field **|B|**, and color contours of χ_sol_ are illustrated for the two cases, without (Fig. 5(a,b)) and with **gradB-**strip (Fig. 5(c,d)) in the xy-plane at z = 160 µm and z = 320 µm. In Fig. 6, the velocity components in all directions are shown at x = 0 versus the y-direction at z = 160 µm (Fig. 6(a,b)) and versus the z-direction at y = 0 (Fig. 6(c,d)) with and without **gradB-**strip. The x- and y-components of the magnetic forces, ${{\bf{f}}}_{{\bf{L}}}$ and ${{\bf{f}}}_{{\boldsymbol{\nabla }}{\bf{B}}}$, versus z at x = 0 are presented in Fig. 7. In the following, the results obtained with and without the **gradB**-strip are discussed separately.

### Velocity field and magnetic forces without the gradB-strip

The simulated MHD flow generated by the Lorentz force ${{\bf{f}}}_{{\bf{L}}}$ is parallel to the electrodes, symmetric across the gap, and fastest in the middle of the gap between the electrodes. This implies that the x-component of the Lorentz force is much stronger than the other force components. From Fig. 7 it can be seen that the x-component has a maximum value of 3.3 N/m³ at the bottom of the cell and decreases with height z. As mentioned before and depicted in Fig. 5(a,b), **|B|** is nearly constant over the cell volume (shown as contours) and oriented perpendicularly to the fluidic chip for z = 160 µm as well as for z = 320 µm. This results in a nearly parabolic shape of the stream wise velocity component u_x_ at x = 0, which has a maximum of about 60 µm/s between the electrodes (Fig. 6(a)). The shape of the horizontal velocity profile between the electrodes depends on the aspect ratio between cell height and electrode distance. The vertical u_x_ profile (Fig. 6(c)) peaks near z = 280 µm, and u_x_ goes to zero at the cell bottom and at the top lid because of the wall friction.

**Figure 8:**
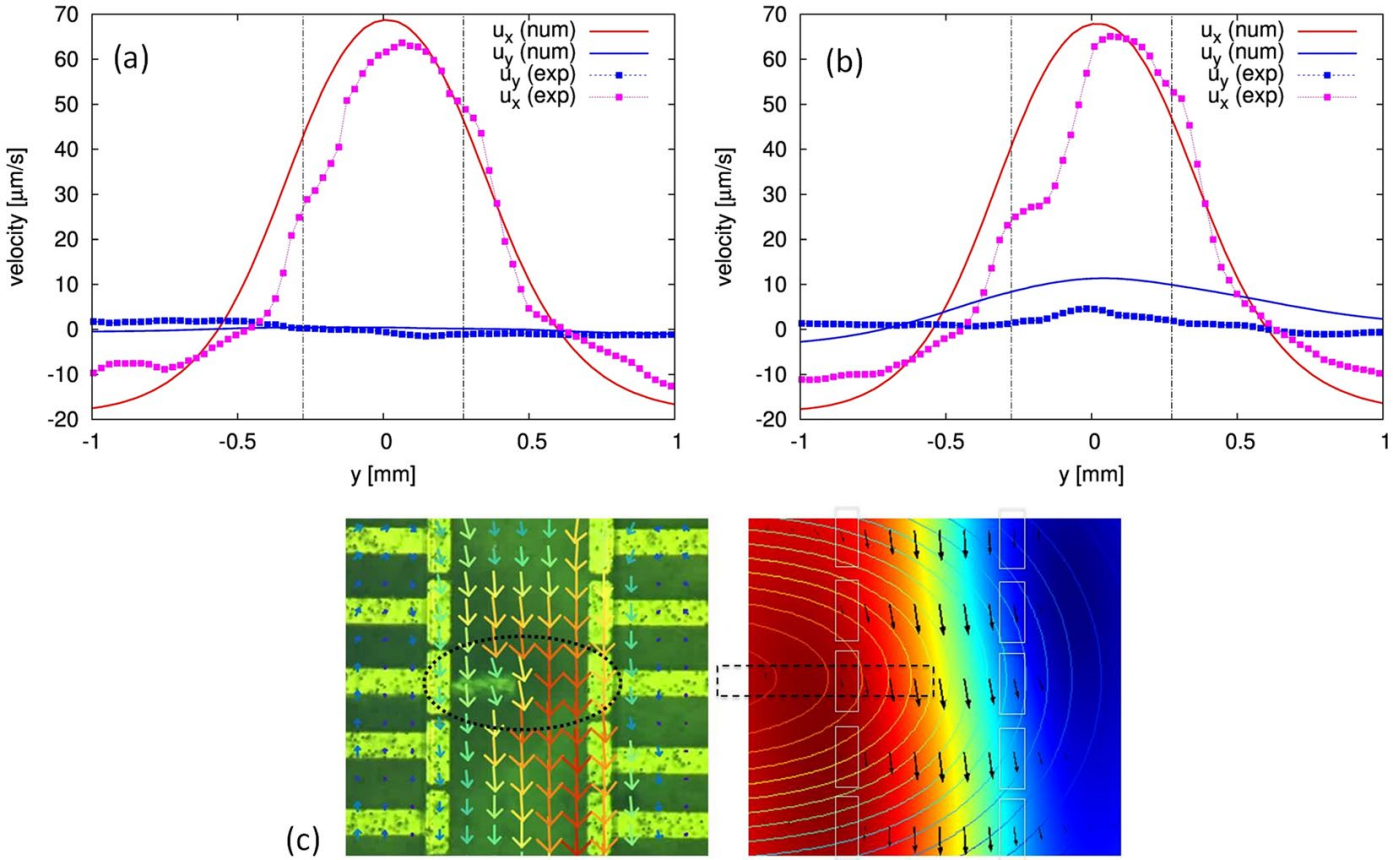
Direct comparison of experimental and numerical velocity data. (**a**,**b**) - Comparison of the x and y-components at x = 0, z = 320 µm versus y-coordinate (crossflow direction) without (**a**) and with (**b**) the **gradB**-strip (the details of the PIV data processing are described in Supplementary Information). (**c**) Zoomed view of the experimental (left) and numerical (right) results near the **gradB**-strip at z = 320 µm.

Compared to other flow directions, the streamwise component u_x_ clearly dominates, in accordance with the measured flow field depicted qualitatively in Fig. 2(a). A more refined velocity analysis of bead motion at x = 0 and z = 320 µm provides a more quantitative comparison with the simulation results and is given in Fig. 8(a). It shows more clearly the u_x_ and u_y_ components, as well as more subtle variations in the flow profile. According to Eq. 2, the species concentration can be related to the spatial distribution of the magnetic susceptibility **χ**_sol_, as shown in Fig. 5(a,b) at t = 60 s, thereby reflecting the already developed diffusion layer due to the electrochemical reaction. The diffusion layer is uniform along the electrodes. From this it follows that, the magnetic field gradient is insignificant and therefore ${{\bf{f}}}_{{\boldsymbol{\nabla }}{\bf{B}}}$ does not contribute to the fluid flow.

### Velocity field and magnetic forces with the gradB-strip

Different from the previous case, from the simulation results shown in Fig. 5(c,d) it is seen that the main flow becomes horizontally deflected near the **gradB-**strip, and that the species distribution is modified accordingly. Looking in more detail at the velocity profile at x = 0, as depicted in Fig. 6(b), the x-component of the velocity u_x_ behaves similar compared to the case without **gradB-**strip, but the horizontal crossflow component u_y_ is threefold larger, reflecting the flow deflection in the region near the **gradB-**strip. From Fig. 6(d) it is seen that the crossflow component u_y_ is maximum at z = 160 µm above the CoFe strip and extends above the level of z = 320 µm. The magnetized CoFe strip causes changes of the magnetic field in its vicinity and creates strong gradients, as discussed above and shown in Figs 3 and 4. However, the resulting changes of the Lorentz force are small and cannot be responsible for the flow deflection, as it was also proven in a separate simulation when the magnetic gradient force ${{\bf{f}}}_{{\boldsymbol{\nabla }}{\bf{B}}}$ was switched off (not shown). Therefore, the effect can be attributed to the magnetic gradient force ${{\bf{f}}}_{{\boldsymbol{\nabla }}{\bf{B}}}$ only. Figure 7 depicts the behavior of different force components versus height at x = y = 0 for the two cases with and without **gradB**-strip. The main driving force for the fluid flow in the gap in both cases is the x-component of ${{\bf{f}}}_{{\bf{L}}}$, which behaves nearly identically in both cases. However, the magnitude of the y-component of ${{\bf{f}}}_{{\boldsymbol{\nabla }}{\bf{B}}}$ significantly increases when the **gradB**-strip is added and reaches about 7.5 N/m³ at the bottom of the chip. Despite the fact that this value is even stronger than the x-component of the Lorentz force ${{\bf{f}}}_{{\bf{L}}}$, the action of the magnetic gradient force ${{\bf{f}}}_{{\boldsymbol{\nabla }}{\bf{B}}}$is limited to the vicinity of the strip only, which results in a local deflection of the main flow that is driven by the Lorentz force ${{\bf{f}}}_{{\bf{L}}}\,$parallel to the electrodes.

In Fig. 8(a,b) a comparison is shown between the refined analysis of the measured and the simulated velocity data at x = 0 at z = 320 µm for the both cases under discussion. The experimental data clearly show the backflow outside the electrode region. The stream-wise components fit well despite a small difference in the peak location and a slight asymmetry in the measured profile, which might be attributed to the experimental conditions mentioned above. Adding the **gradB-**strip for both leads to an increase of the y-component of velocity at the expense of the x-component, the effect is larger in the simulations. Figure 8(c) depicts a cutout of the measured and the simulated flow field in the region of the **gradB-**strip. The flow field obtained by numerical simulation reflects the measured results with high precision.

## Conclusions

A horizontal and nearly uniform redox-MHD flow is generated applying a homogeneous magnetic field oriented perpendicular to an ionic current between two parallel arranged electrodes in a microfluidic cell containing [Fe(CN)_6_]^−3^/[Fe(CN)_6_]^−4^ ions. In this work it is demonstrated that by implementing a movable magnetized ferromagnetic CoFe strip underneath the middle position of the anode the direction of the fluid flow can be changed. [Fe(CN)_6_]^−3^ ions are formed at the anode by the oxidation of [Fe(CN)_6_]^−4^ ions and the fluid with the paramagnetic ions is attracted in regions of high magnetic field gradients near the CoFe strip which leads to modified species distribution. In this region the magnetic field gradient force (**f**_**∇B**_*)* is able to generate a crossflow, which overlays the classical MHD flow driven by the Lorentz force (**f**_**L**_). The direction of the fluid flow above the **gradB**-strip tilts to the cathode, the velocity increases significantly and is lower at the anode. The impact of the magnetic field gradient on the velocity profile is measured by Particle Image Velocity measurements and complemented by 3D numerical simulation. The simulations provide information on **B**, **∇B**, the concentration profile and paramagnetic susceptibility of the fluid, the two magnetic forces, **f**_**L**_ *and* **f**_**∇B**_ as well as the resulting velocity profiles in x, y, z-directions. With this proof-of-concept design, the action of combined magnetic forces has been shown. It is anticipated that with a tailored design of micromagnets and –electrodes a simple method is provided for manipulation of fluids with paramagnetic species. It is feasible to enrich, detect and analyze these species as useful tool for several microbiological, chemical and environmental approaches and applications in lab-on-chip technologies.

## Materials and Methods

### Chemicals and Materials

Chemicals for experiments were all analytical grade and used as received. The sources and grade of all chemicals and materials used for experiments are discussed in the Supplementary Information.

### Microelectrode Chip and gradB-strip

Glass chips (2.54 × 2.54 cm^2^) consisting microelectrodes were used for the MHD experiments. Figure 1(a) shows a photograph of the whole chip with contact pads and leads and Fig. 1(b) shows an enlarged view of the electrode array region. CoFe foils were used as **gradB**-strip. Further details of microelectrode dimensions, fabrication process, and **gradB**-strip are provided in the Supplementary Information.

### The Chip – gradB-strip - Magnet Assembly

The microelectrode chip was connected to an edge connector to control individual electrodes using a galvanostat/potentiostat (CH 760B, CH Instruments, Austin, TX). One array of electrodes (2 to 11) were shorted together as working electrode or anode and the other array (12 to 19) as quasi counter/reference electrode or cathode (two-electrode setup). A PDMS gasket of 760 µm thickness with an open area of 13 × 4.5 mm^2^ define the chamber dimensions and was placed on the microelectrode chip around the electrode region to form an electrochemical cell, leaving the microband electrodes to be exposed to the solution. The experiments were conducted with a solution of 0.1 M K_3_Fe(CN)_6_, 0.1 M K_4_Fe(CN)_6_ and 0.1 M KCl electrolyte. (Control experiments used the supporting electrolyte alone). By adding 20 µL of a polystyrene microbead solution to 1 ml of the solution and using video microscopy, the fluid flow could be monitored. This addition changes the starting solution concentrations to 98% of their original values. Upon pipetting the prepared solution into the opening of the gasket, a glass coverslip was positioned on top to confine the fluid and avoid evaporation. Appropriate precautions were taken during the sealing step to avoid air bubbles in the solution. Then the completed assembly was placed over a NdFeB block magnet on the stage of the microscope. The **gradB**-strip was inserted between the chip and magnet in a way such that the CoFe strip was positioned directly in the middle of the 5^th^ electrode in the array (Fig. 1(b)).

### MHD Pumping under Controlled Current Conditions and Flow Profile Analysis

An anodic current of −10 µA applied between the two arrays, resulted in a primary fluid flow driven by MHD along the gap between the anode and cathode arrays and in the direction as expected by eq. 1. The fluid flow was investigated at various positions of the **gradB**-strip under the microelectrode chip, by gradually moving it horizontally in a direction perpendicular to the electrode arrays and from the anode side to the cathode side, across the 550 µm gap (Fig. 2(a–h)). Fluid flow was recorded with a Sony camcorder (30 frames/s) mounted on the microscope (Leica 2500 DM, Leica Biosystems Inc., IL) for particles in focus at 160 µm and 320 µm above the chip surface. The video recording length for each experiment was 1 min 30 s. For the first 15 s, the cell was at open circuit. For the next 60 s, an anodic current of −10 µA was applied between the two sets of electrodes. The current was then switched off for the last 15 s. The videos were analyzed with Particle Imaging Velocimetry (PIV) software and the resulted velocity profiles were corrected for a slight unavoidable tilt in the images and averaged in the flow direction to yield the refined profiles given in Fig. 8(a,b). Detailed information on the applied analysis methods and error analysis are given in the Supplementary Information.

### COMSOL simulation

Steady numerical simulations were performed by the commercial software COMSOL Multiphysics 5.2a to obtain the spatial distribution of the magnetic field and to solve the coupled problem of mass transfer and convection under the influence of the magnetic forces. A sketch of the simulation setup is shown in SI Fig. S6. The microfluidic cell, consisting of the electrode arrays at the chip surface is defined by a PDMS cut-out of 13 × 4.5 × 0.76 mm³ dimension. The electrode height was neglected for simplification. The distribution of the magnetic induction **B**, results from the cuboid NdFeB magnet and optionally the CoFe strip. The magnetic field induced by the galvanic current can be neglected. The calculation of **B** was performed in a large spherical domain of diameter 80 mm with the magnet in its center to exclude the influence of the boundary condition of magnetic isolation B_n_ = 0. The equations to be solved are

4 $${\bf{H}}=-\,{\rm{\nabla }}{V}_{m},\,{\rm{\nabla }}\cdot {\bf{B}}=0$$

where *V*_*m*_ defines the magnetic potential of the magnetic field strength **H**. The relations between **B** and **H** are as follows: for the NdFeB magnet, it holds **B** = µ_0_µ_r_ **H** + **B**_**r**_, where µ_0_, µ_r_ and **B**_**r**_ denote the permeability constant, the relative permeability and the remanent magnetic flux density of the magnet. µ_r_ and **B**_**r**_ are set to 1 and 1.28 T in z-direction, respectively. For the CoFe strip, based on own measurements, a nonlinear magnetization curve $B=f(|H|)\frac{H}{|H|}$ with a saturation magnetization of about 2.22 T is applied (SI Fig. S3). For the microfluidic cell and the surrounding air volume, **B** = µ_0_µ_r_ **H** holds.

For obtaining the ionic current density **j**(**r**) in the microfluidic cell, a constant electrical conductivity (σ) of the electrolyte is assumed, and a Laplace equation for the electric potential Φ is solved:

5 $${\rm{\Delta }}{\rm{\Phi }}=0,\,{\bf{j}}=-\,\sigma \,{\rm{\nabla }}{\rm{\Phi }}.$$

The potential difference between electrode arrays was adjusted to obtain the desired −10 µA cell current. At insulating walls, a zero Neumann condition for the normal component of Φ applies.

The convection of the electrolyte in the microfluidic cell (velocity field **u**) was determined by the steady incompressible Navier-Stokes equation with the two magnetic forces and the incompressibility constraint:

6 $$\rho ({\bf{u}}{\rm{\nabla }}){\bf{u}}=-\,{\rm{\nabla }}p+\eta {\rm{\Delta }}{\bf{u}}+{{\bf{f}}}_{{\bf{L}}}+{{\bf{f}}}_{{\rm{\nabla }}{\bf{B}}},\,{\rm{\nabla }}\cdot {\bf{u}}=0.$$

Here, *p*, *ρ* and *η* denote the pressure field, the density and the dynamic viscosity of the electrolyte. Buoyancy effects are neglected so far. The Lorentz force (1) and the magnetic gradient force (2) vary in space. In detail, (2) depends on the local value of the magnetic susceptibility χ (r) of the solution determined by the concentration of the [Fe(CN)]^3−^ species χ = χ_H2O_ + c_[Fe(CN)6]_^3−^· χ _[Fe(CN)6]_^3−^, χ _[Fe(CN)6]_^3−^ = 2.65·10^−8^ m³/mol. The diamagnetic susceptibility of the second redox species can be neglected. Concerning the boundary conditions, a no-slip condition is applied at all walls, electrodes, and at the upper lid. Despite the galvanostatic condition, the experiment finally leads to diffusion-controlled mass transfer. The velocity measurements were performed during a duration of 60 s after the current was switched on. It can be estimated that this time is too short for a steady flow regime to develop. Therefore, in order to approximate the experiment in a steady simulation, the amplitude of the magnetic gradient force was reduced to 10%.

Finally, for the mass transfer of the [Fe(CN)_6_]^3−^ species a convection-diffusion equation is solved for:

7 $$({\bf{u}}\nabla )c=D{\rm{\Delta }}c$$

here, D = 1·10^−9^ m²/s denotes the coefficient of molecular diffusion. As a limiting-current regime was assumed at the electrodes, constant values of the concentration (0 and 2·c_0_ at cathode and anode, respectively) were set. The bulk concentration c_0_ was set to 0.1 M.

As species are advected with the velocity field and the magnetic gradient force depends on species concentration, a strong coupling of convection and mass transport, eqs (6) and (7), exists. We also neglected the possible Joule heating effect of the electrolyte.

## Supplementary information

Supplementary Information: Combining magnetic forces for contactless manipulation of fluids in microelectrode-microfluidic systems

## Supplementary information

**Supplementary information** accompanies this paper at 10.1038/s41598-019-41284-0. The datasets generated during and analyzed during the current study are available from the corresponding author on reasonable request.
