# Supplementary material for: Combining magnetic forces for contactless manipulation of fluids in microelectrode-microfluidic systems
Source: Sci Rep. 2019 Mar 25;9:5103. doi: 10.1038/s41598-019-41284-0 (PMC6433926; doi:10.1038/s41598-019-41284-0)
Supplement: Supplementary file 1 — Supplementary Information: Combining magnetic forces for contactless manipulation of fluids in microelectrode-microfluidic systems [file 41598_2019_41284_MOESM1_ESM.docx]

**Supplementary Information:**

**Combining magnetic forces for contactless manipulation of fluids in microelectrode-microfluidic systems**

Veronika Haehnel^1^, Foysal Z. Khan^2^, Gerd Mutschke^3^, Christian Cierpka^4^, Margitta Uhlemann^1^, Ingrid Fritsch^2^

***Electrochemical characterization***

Figure S1a shows four cyclic voltammetry (CV) responses of the redox couple in a solution of 0.1 M K_3_Fe(CN)_6_ and 0.1 M K_4_Fe(CN)_6_ in 0.1 M KCl as supporting electrolyte to determine suitable currents for subsequent microfluidics experiments that involve galvanic control of the electrodes in the magnetic fields. A two-electrode setup similar to that used in the microfluidics studies was used for CV, where the two parallel arrays, each of nine shorted electrodes, act as anode and cathode. The CV response exhibits a shape that is typical for diffusion-limited behavior. The peak separation is ~200 mV, which is 140 mV more than the usual ~60 mV for a reversible, one-electron redox reaction. This indicates the presence of an uncompensated resistance, R_u_, of about 2000 Ohm based on the peak current of 35 µA and an iR_u_ drop of 70 mV beyond the expected 30 mV when swept in a single direction. This R_u_ is not unusual for electrodes formed from a thin conducting film and a narrow solution cross section. To avoid heating and gas bubble formation the current applied for the pumping studies was set below the mass transfer limit, to the anodic current of -10 µA for all addressed electrodes. In Figure S1b the potential transients recorded for the three different galvanic experiments are depicted: (black) without applied magnetic field, (blue) with the NdFeB permanent magnet placed behind the microfluidic chip and (red) with the additional magnetic field gradient template. The mean and the standard deviation of the potential at t = 60 s was determined from 12 runs. Independent of whether a magnetic field was applied or not the mean was 68 mV ± 1 mV. The thickness of the diffusion layer (δ =$\sqrt{Dt}$; D = 7.84 x 10^-6^ cm²/s)^[1](#_ENREF_1)^ after 60 s is about 385 µm. Because the gap between the parallel electrode arrays is 550 µm, an overlap of the diffusion layer for both processes at the anode and cathode is expected for a stationary solution without applied magnetic field. We showed previously with a similar chip design that spacings of as much as 407 µm between similarly biased, activated electrodes within the anode and cathode arrays will still propel fluid along a straight path in the gap between them.^[2](#_ENREF_2)^

***Chemicals and Materials***

Chemicals for experiments were all analytical grade and used as received. Electrolyte and redox solutions were prepared with 18 MΩ cm deionized water from Ricca Chemical Company (Arlington, TX). K_3_Fe(CN)_6_ and K_4_Fe(CN)_6_ were obtained from EM Science, Gibbstown, NJ and J.T. Baker, Phillipsburg, NJ, respectively. KCl (99% assay) and pre-cleaned micro cover glass (24 X 50 mm^2^) were purchased from VWR International LLC (West Chester, PA). The 0.40 T nickel coated NdFeB block permanent magnet (25.4 × 25.4 × 12.7 mm^3^) grade N45, Q500H was acquired from Amazing Magnets, Irvine, CA. Polystyrene latex microspheres (10 µm), stabilized with a slight anionic charge from surface sulfate groups (2.5 wt% dispersion in water), were obtained from Alfa Aesar (Ward Hill, MA).

Pyrex (glass) wafers (125 mm diameter and 550−600 μm thick) were purchased from Mark Optics (Santa Ana, CA). Edge connectors (solder contact, 20/40 position, and 0.05 in. pitch) were acquired from Sullins Electronics Corp. (San Marcos, CA). Poly-(dimethylsiloxane), PDMS, gaskets were fabricated by using a silicon elastomer base, curing agent, and OS-30 solvent (Ellsworth Adhesives, Milwaukee, WI) as described previously.^[3](#_ENREF_3)^


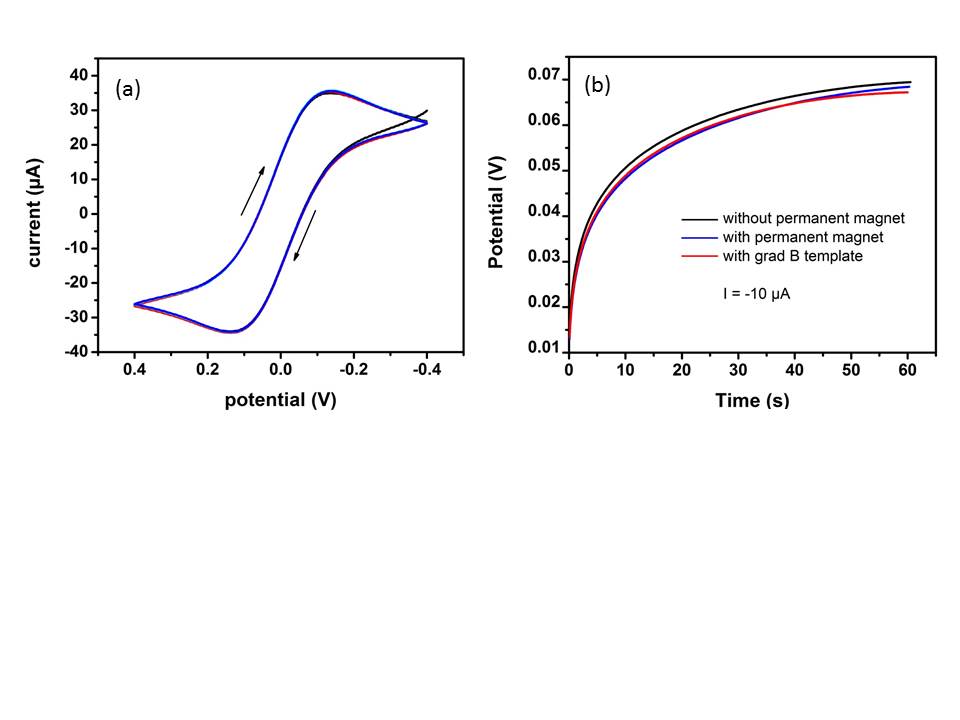


**Figure S1:** (a) Cyclic voltammogram of 0.1 M K_3_Fe(CN)_6_ / 0.1 M K_4_Fe(CN)_6_ and 0.1 M KCl, dE/dt = 20 mV/s, (b) chronopotentiometric plot with an applied anodic current of -10 µA, in the absence and presence of a permanent magnet and in the presence of both the permanent magnet and the **gradB**-strip

***Microelectrode Chip***

Glass chips (2.54 × 2.54 cm^2^) consisting of two, parallel, linear arrays of nine, individually-addressable gold microelectrodes were fabricated using photolithography as previously reported, shown in Figure S2.^[2](#_ENREF_2)^ Each electrode is 252 µm long, 97 µm wide, approximately 100 nm high, and separated by a gap of 23 µm from adjacent electrodes. The 550 µm gap between the two linear arrays of electrodes serves as the pumping region. Contact pads at one end of the chip were used to make the connection between each electrode and the potentiostat. Benzocyclobutene, an insulating polymer covered the chip except over the electrodes and contact pad areas to isolate the leads. Two continuous electrode strips could have been used instead. However, this design was available in our laboratory on a transparent substrate, which allowed visual placement of the CoFe strip beneath the electrodes.

**
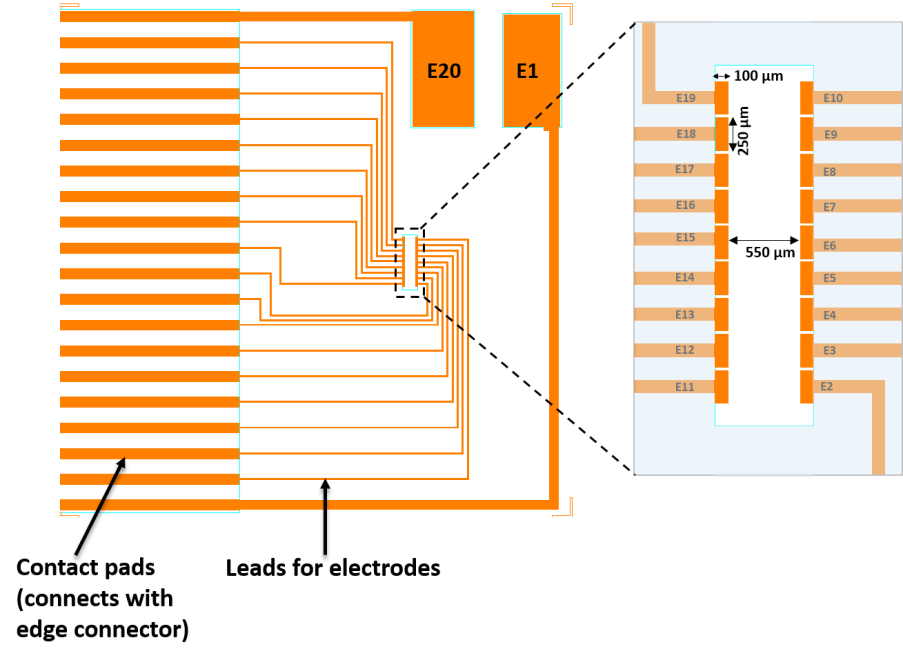
**

**Figure S2:** (left) the schematic sketch of the chip with the contact pads and leads for electrodes and (right) the enlarged area of the electrodes with dimensions. The CoFe strip is placed underneath the electrode E6.

***The gradB-strip***

CoFe foils purchased as Permendur 49® (Co49/Fe49/V2 – Goodfellow) with a thickness of 50 µm were cut into strips (several mm in length, 2.5 mm in height). The strips of CoFe were embedded in epoxy (EpoFix –Resin, Struers) in circular “master forms”. After 24 h, their height was reduced to approximately 0.97 mm by cutting and grinding and finished by polishing. We refer to this single CoFe strip embedded in circular epoxy material as the “**gradB-**strip”. The saturation magnetization of CoFe used in simulation amounts to be about 2.22 T at room temperature. It was determined by employing the Vibrating Sample Magnetometer (Quantum design PPMS, 9 T), Figure S3.


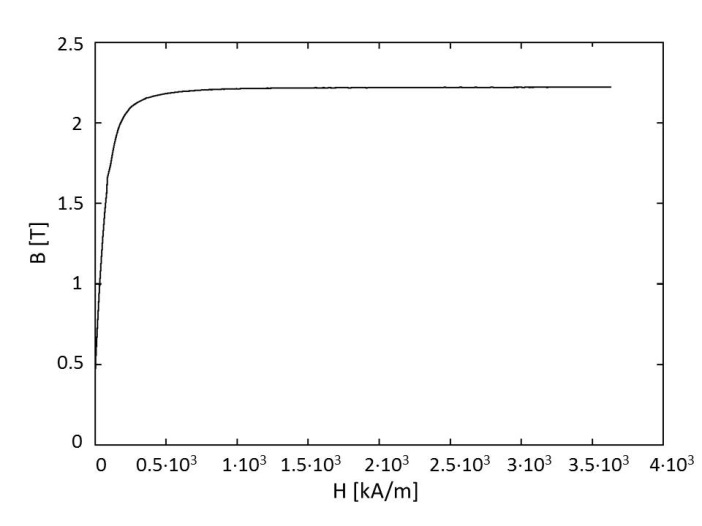


**Figure S3**: Magnetization curve for the CoFe-strip material at room temperature

It can be seen in Figure S4 that the magnitude of the magnetic field gradient (B gradB) decays exponentially with growing distance from the surface of the CoFe-strip. Since the thickness of the bottom part of the chip was fixed, the dimensions of the CoFe strip were chosen such as to have a sufficiently strong magnetic field gradient inside the cell.


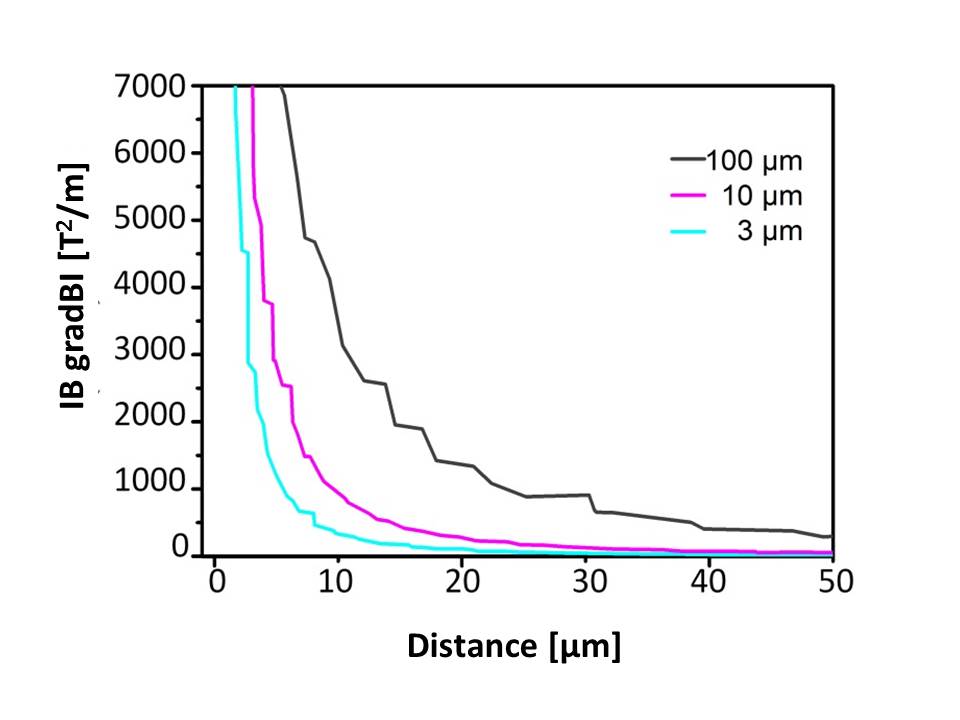


**Figure S4:** Magnetic field gradient dependent on the distance from the surface and the thickness of the CoFe strip.

***Particle Imaging Velocimetry (PIV) Analysis***

The horizontal translation of the microbeads was analyzed in two ways. For the 2D vector plots cross correlation PIV (Dynamic Studio, v. 3.00, Dantec Dynamics, Copenhagen, Denmark) was done based on images extracted from the video recording by maintaining original resolution and aspect ratio. Every 10^th^ frame was then loaded into the PIV software, and the bead movement in that sequence was analyzed. Image preprocessing consists of subtracting the mean intensity over all images, maintaining proper image contrast, and interpolating the group into (N-1) double frames. Finally, the 2D2C vector field was evaluated by using averaging in the correlation plane of these double frames, which is known to give reliable data especially in microfluidics.

In order to increase the spatial resolution for the velocity profiles presented in Figure 8 the data processing for the profiles contained the following steps. ^[4](#_ENREF_4)^ To enable a robust and reliable estimation of the velocity the bright field images were inverted. To remove the electrode structures from the images that would disturb the further processing an average image was subtracted. Since the particle displacement was very small, each image was correlated with its 30^th^ successor to give a mean displacement in the order of 10 pixels. As in the previous data analysis ensemble correlation was used to allow for a higher spatial resolution and give reliable date in the case of low seeding concentrations as typical for micro PIV. Finally, multi-pass multi-grid ensemble correlation was performed over a set of 1,700 images with a final interrogation window size of 8x8 pixel with 50% overlap which gives a spatial resolution of about 14 µm in each direction. The velocity profiles were corrected for a slight unavoidable tilt in the images and averaged in flow direction.

As can be seen in Figure S5, the standard deviation of the velocity during the experiments (z = 320 µm) amounts to 5.1 µm/s (dotted lines) and corresponds to 12% of the mean. However, this relatively large value is mainly caused by the low seeding concentration. There exist regions, where too little particles are present for a valid detection of the velocity vector. So the standard deviation rather corresponds to spatial limitation of the measurement technique than to a fluctuating velocity in the channel.


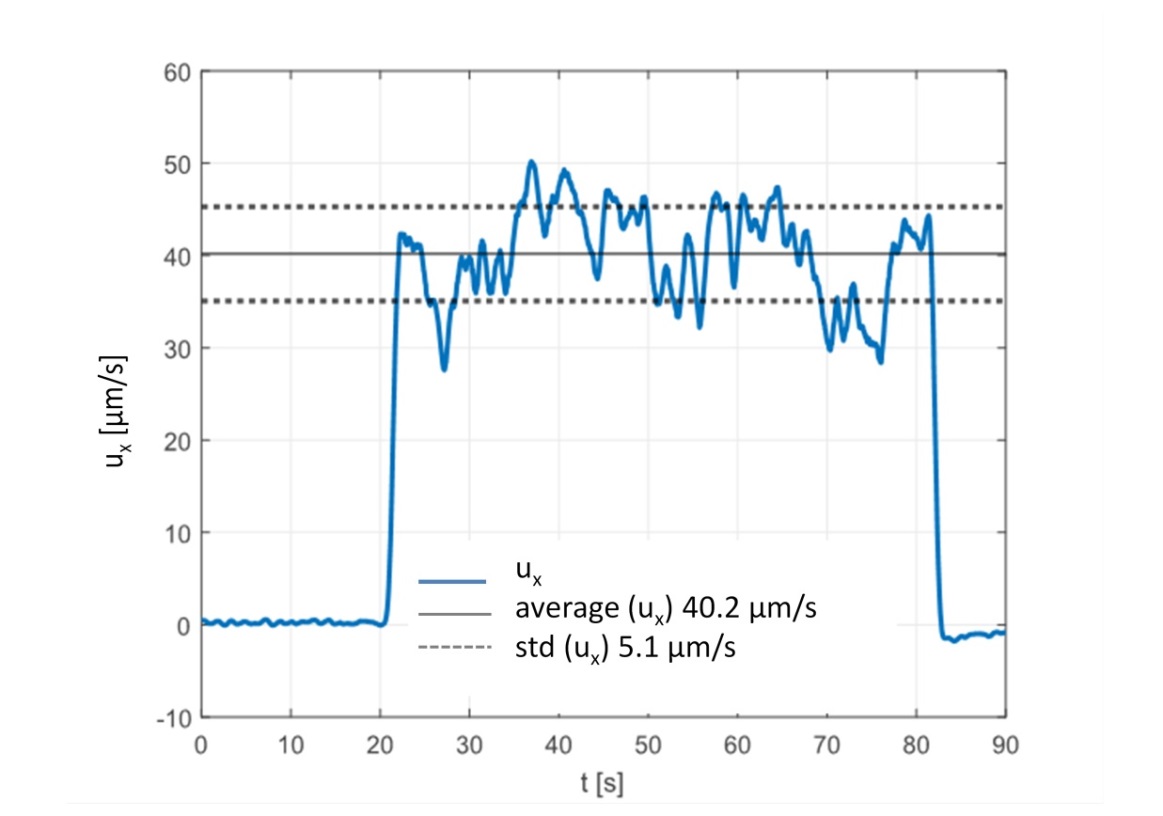


**Figure S5:** Mean and standard deviation for the u_x_ of the velocity above the **gradB**-strip at z = 320 µm.

***Simulation details***

Due to the large span in length scales (electrode tiles, flow cell, magnetic domain), in order to accurately resolve all geometric details, the finite element grid generated and used for the simulations contained about 3.2 Mio. elements and was iteratively and carefully refined in regions where large gradients of the solution occur. The two arrays of a set of 9 small electrodes (each is 252 μm x 97 μm and spaced from the others by 23 µm, Figure 1) were treated as one electrode at each side in the numerical setup. The simulation setup is sketched in Figure S6.

**
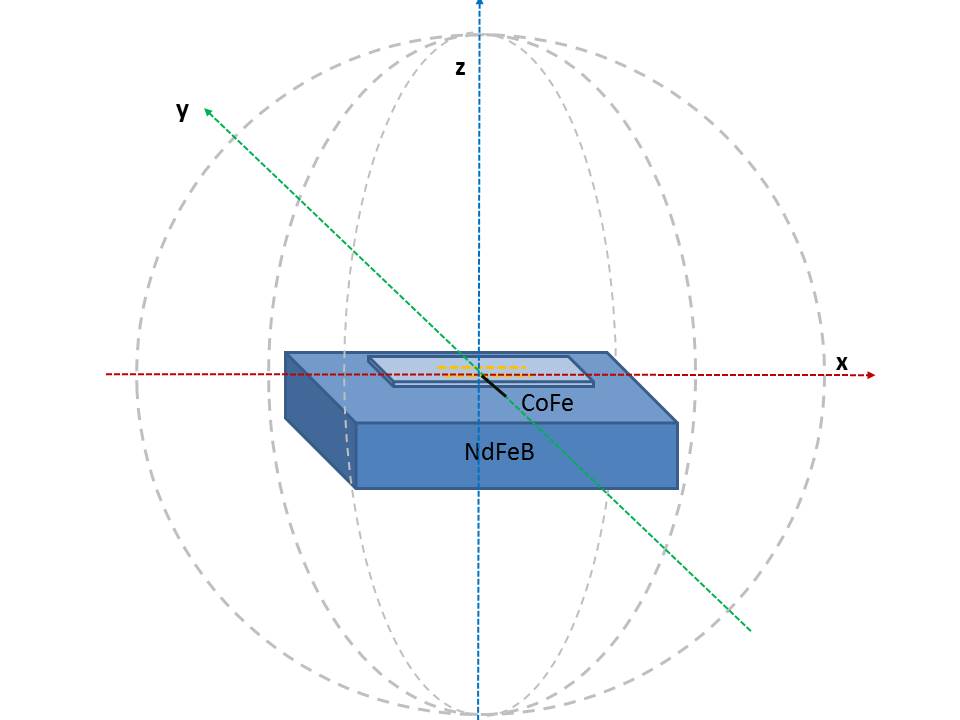
**

**Figure S6**: Sketch of the simulation setup with the permanent magnet, the CoFe strip and the microfluidic chip with the array of Au-electrodes. Dotted lines represent the sphere of d=80 mm for the simulation grid and the axis of the coordinates (not drawn to scale).

**References**

1 Scrape, P. G., Gerner, M. D., Weston, M. C. & Fritsch, I. Redox-Magnetohydrodynamics for Microfluidic Control: Remote from Active Electrodes and Their Diffusion Layers. *Journal of the Electrochemical Society* **160**, H338-H343, doi:10.1149/2.076306jes (2013).

2 Weston, M. C., Nash, C. K., Homesley, J. J. & Fritsch, I. Maximizing Flow Velocities in Redox-Magnetohydrodynamic Microfluidics Using the Transient Faradaic Current. *Analytical Chemistry* **84**, 9402-9409, doi:10.1021/ac302063a (2012).

3 Sahore, V., Kreidermacher, A., Khan, F. Z. & Fritsch, I. Visualization and Measurement of Natural Convection from Electrochemically-Generated Density Gradients at Concentric Microdisk and Ring Electrodes in a Microfluidic System. *Journal of the Electrochemical Society* **163**, H3135-H3144, doi:10.1149/2.0181604jes (2016).

4 Cierpka, C. & Kähler, C. J. Particle imaging techniques for volumetric three-component (3D3C) velocity measurements in microfluidics. *Journal of Visualization* **15**, 1-31, doi:10.1007/s12650-011-0107-9 (2012).
